# Role of receptor for advanced glycation end products (RAGE) in liver disease

**DOI:** 10.1186/s40001-015-0090-z

**Published:** 2015-02-11

**Authors:** Sho-ichi Yamagishi, Takanori Matsui

**Affiliations:** Department of Pathophysiology and Therapeutics of Diabetic Vascular Complications, Kurume University School of Medicine, 67 Asahi-machi, Kurume, 830-0011 Japan

**Keywords:** AGEs, RAGE, NASH, HCC, Liver injury

## Abstract

Receptor for advanced glycation end products (RAGE) belongs to a immunoglobulin superfamily of cell surface molecules that could bind to a number of ligands such as advanced glycation end products, high-mobility group protein box-1, S-100 calcium-binding protein, and amyloid-β-protein, inducing a series of signal transduction cascades, and being involved in a variety of cellular function, including inflammation, proliferation, apoptosis, angiogenesis, migration, and fibrosis. RAGE is expressed in hepatic stellate cells and hepatocytes and hepatoma cells. There is accumulating evidence that engagement of RAGE with various ligands elicits oxidative stress generation and subsequently activates the RAGE downstream pathway in the liver, thereby contributing to the development and progression of numerous types of hepatic disorders. These observations suggest that inhibition of the RAGE signaling pathway could be a novel therapeutic target for liver diseases. This article summarizes the pathological role of RAGE in hepatic insulin resistance, steatosis and fibrosis, ischemic and non-ischemic liver injury, and hepatocellular carcinoma growth and metastasis and its therapeutic interventions for these devastating disorders.

## Review

### Introduction

Receptor for advanced glycation end products (AGEs) (RAGE) is a multiligand cell surface receptor that belongs to the immunoglobulin superfamily [1-4]. RAGE is a pattern recognition receptor with a molecular mass of 47 to 55 kDa, consisting of an extracellular region made of V1-, C1-, and C2-type immunoglobulin domains, transmembrane-spanning domain, and a short cytosolic tail [1-4]. V1- and C1-type domains are considered as the principal interacting sites for various ligands, whereas cytosolic tail is essential for downstream signaling pathway of RAGE [1-4]. V1- and C1-type domains of RAGE have a net positive charge that might act as an electrostatic trap for negatively charged macromolecules such as AGEs, high-mobility group protein box-1 (HMGB1), S-100 calcium-binding protein, and amyloid-β-protein [1-4].

RAGE is found in an oligomeric or preassembled state within the plasma membrane [1-4]. RAGE expression is usually low in the majority of healthy adult tissues, but its levels are elevated under pathological conditions such as diabetes, cardiovascular disease, Alzheimer’s disease, and cancer [4-7]. Multimeric ligands could stabilize the assemblies of RAGE and shift the equilibrium to larger oligomers [1-4]. Moreover, engagement of RAGE with RAGE ligands increases expression of RAGE itself in a variety of cells [4-7]. These positive feedback loops could partly explain why RAGE-ligands interaction could lead to sustained activation of the RAGE downstream pathway.

RAGE activation by various ligands have been reported to increase oxidative stress generation and subsequently evoke inflammatory, proliferative, angiogenic, fibrotic, thrombogenic, and apoptotic reactions in numerous cell types via activation of diverse intracellular signaling pathways such as nuclear factor-κB (NF-κB), mitogen-activated protein kinase (MAPK), Janus kinase-signal transducers and activators of transcription (JAK-STAT), and phosphoinositol 3 kinase [8-10]. RAGE is expressed in hepatic stellate cells and hepatocytes and hepatoma cells [11]. There is accumulating evidence that activation of RAGE signaling pathways in the liver could contribute to the development and progression of numerous types of hepatic disorders [11,12]. These observations suggest that inhibition of the RAGE downstream pathway could be a novel therapeutic target for various liver diseases. This article summarizes the pathological role of RAGE in hepatic insulin resistance, steatosis and fibrosis, ischemic or non-ischemic liver injury, and hepatocellular carcinoma (HCC) growth and metastasis and its therapeutic interventions for these devastating disorders.

In the present review, literature searches were undertaken in Medline by the PubMed interface. Non-English language articles were excluded. Key words (RAGE) and (review or liver or hepatic) have been used to select the articles.

#### Hepatic insulin resistance, steatosis, and fibrosis

1. Hepatic insulin resistance
  Non-alcoholic fatty liver (NAFL) is the most common chronic liver disease in the world [13-16]. NAFL is characterized by hepatic steatosis in the absence of significant alcohol intake or other known liver diseases. NAFL includes a wide spectrum of liver diseases, ranging from fatty liver, a benign and non-progressive condition, to non-alcoholic steatohepatitis (NASH), a potentially progressive disease that may cause cirrhosis, liver failure, and HCC [13-16]. NASH is considered the hepatic manifestation of the metabolic syndrome and is associated with central obesity, insulin resistance, diabetes, essential hypertension, and dyslipidemia [17,18].
  We have previously shown that serum levels of AGEs are elevated under oxidative stress, inflammatory, and/or diabetic conditions and correlated with insulin resistance and decreased adiponectin levels, thereby being one of the useful biomarkers for differentiating NASH from simple steatosis [19-24]. Further, activation of RAGE downstream pathway by AGEs evokes inflammatory reactions and impairs insulin signaling in Hep3B hepatoma cells by stimulating c-Jun NH2-terminal kinase (JNK)- and IκB kinase-dependent serine phosphorylation of insulin receptor substrate-1 via Rac-1 activation [25-27]. Combination therapy with nateglinide, a rapid-onset/short-duration insulinotropic agent and telmisartan, an angiotensin II type 1 receptor blocker with partial agonistic activity of peroxisome proliferator-activated receptor-γ (PPARγ), improves hepatic insulin resistance in Zucker fatty rats by suppressing the AGE-RAGE axis as well [28]. These observations suggest the involvement of AGE-RAGE axis in inflammation and insulin resistance in the liver.
2. Hepatic steatosis
  High AGE-containing methionine choline-deficient (MCD) diet increased AGE contents, lipid peroxidation product, 4-hydroxynonenal levels, and NADPH oxidase-driven superoxide generation in the liver of Sprague Dawley (SD) rats compared to SD rats with MCD diet alone, which were associated with severity of steatohepatitis and hepatic fibrosis in these animals [29]. Furthermore, AGEs significantly increased reactive oxygen species (ROS) production, RAGE, monocyte chemoattractant protein-1 (MCP-1), interleukin-6 (IL-6), and α-smooth muscle cell actin expression in hepatic stellate cells (HSCs) derived from MCD diet-fed rats [29]. In addition, Gaen et al. recently reported that carboxymethyllysine (CML) accumulation in the liver of obese individuals was associated with hepatic pro-inflammatory gene expression as well as grade of steatosis and steatohepatitis [30]. They also found that fatty acids could stimulate CML accumulation in hepatocytes and subsequently elicit inflammatory reactions via RAGE induction [30]. Lack of galectin-3, a scavenging receptor for AGEs, has been shown to progress the NAFL disease in mice, which was also associated with enhanced hepatic accumulation of AGEs and RAGE expression [31,32]. These findings suggest that dietary glycotoxins and/or lipid peroxidation-induced AGE accumulation in the liver might promote the progression of NAFL to NASH by enhancing the RAGE-mediated inflammatory reactions.
3. Hepatic fibrosis
  HSCs are the main extracellular matrix-producing cells in the liver and thus play a pivotal role in liver fibrosis [33]. Fehrenbach et al. showed that expression of RAGE was up-regulated during the process of transdifferentiation of HSCs to myofibroblasts (MFB) and transforming growth factor-β1 (TGF-β1) increased RAGE and α-smooth muscle actin levels at filopodial membranes of MFB, thus suggesting a role of TGF-β1-RAGE axis in the spreading and migration of activated HSCs. They also showed that ligand activation of RAGE increased ROS formation and subsequently induced MAPK and NF-κB signaling pathways in HSCs [33]. Furthermore, we have found that AGE-RAGE interaction induces proliferative, inflammatory, and fibrotic reactions in HSCs by stimulating TGF-β1, MCP-1, collagen type I alpha2, and α-smooth muscle actin expression via NADPH oxidase-derived ROS generation [34]. ROS generation in HSCs evoked by the AGE-RAGE axis is blocked by an inhibitor of Rac-1, a component of NADPH oxidase, or p47phox silencing [35]. These observations suggest that RAGE-mediated, NADPH oxidase-derived ROS could contribute to hepatic fibrosis via the activation of HSCs.
  Curcumin, a main curcuminoid present in turmeric, a popular Indian spice, inhibited AGE-induced RAGE expression in HSCs by increasing PPARγ activity and stimulating *de novo* synthesis of glutathione, which could lead to the suppression of oxidative stress generation, inflammation, and HSCs activation [36]. Curcumin was also shown to eliminate the deleterious effects of AGE-RAGE axis on HSCs by inducing gene expression of AGE receptor-1, a responsible receptor for detoxification and clearance of AGEs, partly via interruption of leptin signaling and activation of transcription factor NF-E2 p45-related factor 2 [37,38]. We have previously shown that (1) pigment epithelium-derived factor (PEDF), a glycoprotein with anti-oxidative, anti-inflammatory, and PPARγ-stimulating properties blocks the AGE- or IL-6-induced hepatic inflammation and (2) serum PEDF levels are independently associated with procollagen type III N-terminal peptide, a marker of hepatic fibrosis in patients with NAFL as a counter system against insulin resistance-related metabolic derangements [39-44]. Stimulation of PPARγ may be a therapeutic target for preventing the HSCs activation.
  In normal rats, chronic AGEs administration induced significant increases in α-smooth muscle actin levels, but did not induce fibrosis or biochemical evidence of liver injury [45]. However, injection of AGEs to rats following bile duct ligation significantly increased hepatic fibrosis, which was in association with oxidative stress and RAGE overexpression in the liver [45]. Furthermore, RAGE gene-silencing therapy decreased serum levels of inflammatory cytokines, reduced hepatic levels of α-smooth muscle actin and collagen I, markers of HSCs activation, and improved inflammatory activity grade and fibrosis stage of CCl4-induced liver injury in rats [46]. Kao et al. reported the involvement of HMGB1 released from damaged hepatocytes and its interaction with RAGE in the pathogenesis of HSCs activation and liver fibrosis [47]. Carotenoids and polyphenols present in peach-derived products have been shown to attenuate the CCl4-induced oxidative stress and liver damage by suppressing RAGE expression [48].

#### Ischemic or non-ischemic liver injury

##### Ischemic liver disease

Hepatic ischemia/reperfusion (I/R) injury associated with liver transplantation and hepatic resection is characterized by hepatocyte damage and enhanced inflammatory reactions [49]. Administration of soluble form of RAGE (sRAGE) has been reported to increase survival of mice after hepatic I/R injury by suppressing the RAGE downstream pathway, which was associated with decreased cell death and necrosis of hepatocytes as well as increased proliferative activity of liver cells [49]. MAPK, JNK, and JAK-STAT were activated in I/R-injured liver, while NF-κB was suppressed. All of these changes were ameliorated by the treatment with sRAGE, in parallel with increased expression of pro-regenerative cytokine [49]. In addition, RAGE-mediated increased expression of early growth response-1 (Egr-1), an inducible zinc finger transcription factor activated in response to cell stress, was involved in enhanced inflammatory reactions in the I/R-injured liver [50]. Losartan, a blocker of angiotensin II type 1 receptor, inhibited the I/R injury-induced hepatocyte apoptosis and inflammation by suppressing the RAGE expression and subsequent activation of Egr-1 via PPARγ activation [51]. These findings suggest that RAGE could modulate hepatic I/R injury, at least in part by activation of key signaling pathways linked to pro-inflammatory and cell death-promoting responses.

#### Non-ischemic liver disease

Uncoupling protein-2 (UCP2) knockout mice showed higher malondialdehyde levels and reduced glutathione/glutathione disulfide ratios as well as significantly higher hepatic levels of AGEs and RAGE compared with normal mice [52]. Galactosamine/lipopolysaccharide (G/L)-induced liver injury was enhanced in UCP2 knockout mice, which was associated with increased AGEs and RAGE levels in the liver [52]. Further, aging accelerated the harmful effects of UCP2 deficiency on AGE-RAGE axis and G/L-induced liver injury by suppressing hepatic activity of glyoxalase-I, a detoxifying enzyme for methyglyoxal, one of the precursors of AGEs [53]. sRAGE treatment has been found to significantly diminish liver damage and increased survival particularly in both young and old UCP2 knockout mice [52,53]. These observations suggest that mitochondrial dysfunction-associated oxidative stress could activate hepatic AGE-RAGE axis, leading to augmentation of inflammation-induced liver injury. Anti-RAGE antibody therapy inhibited the G/L-induced acute liver injury in senescence-accelerated-prone mice [54]. Sinusoidal perfusion failure and inflammation in the livers exposed to G/L were also suppressed by the treatment with anti-RAGE antibody [55]. These findings could further support the clinical relevance of RAGE blockade for the treatment of endotoxemic liver damage.

RAGE is up-regulated in liver remnants selectively after massive (85%) versus partial (70%) hepatectomy, principally in mononuclear phagocyte-derived dendritic cells [56]. Furthermore, blockade of RAGE, using pharmacological antagonists or transgenic mice in which a signaling-deficient RAGE mutant is expressed in cells of mononuclear phagocyte lineage, significantly increases survival after massive liver resection [56]. RAGE inhibition induced NF-κB activation and resultantly enhanced expression of regeneration-promoting cytokines in the massively injured liver [56]. sRAGE treatment also decreased hepatic necrosis and inflammatory and oxidative stress reactions and increased survival of mice with acetaminophen-induced liver injury [57]. Blockade of RAGE may improve survival of mice with extensive hepatectomy by restoring adaptive mechanisms triggered by Myd88 signaling pathways [58].

#### HCC

RAGE was expressed in human HCC cell line, Hep3B and HepG2 cells, whereas AGEs increased vascular endothelial growth factor (VEGF) expression in these cell types [59,60]. Furthermore, AGE-treated conditioned medium significantly increased proliferation, migration, and tube formation of endothelial cells (ECs), thus suggesting that AGE-RAGE signaling could enhance the angiogenic potential of HCC cells by up-regulating VEGF expression [59]. AGEs have been shown to increase the growth of HuH7, other type of HCC cell line as well [60]. MK615, an extract from Japanese apricot was reported to inhibit the AGEs-induced proliferation of HuH7 by suppressing RAGE expression [60]. Orally administered high-AGE beverage induced hepatic VEGF expression and AGE accumulation in rats, suggesting a pathological role of dietary AGEs for the progression of HCC [61].

We have previously shown that AGE-RAGE interaction-mediated, NADPH oxidase-induced ROS generation stimulates proliferation and tube formation of ECs, the key steps of tumor angiogenesis, through VEGF expression via transcriptional activation of NF-κB and activator protein-1 [62-66]. Furthermore, activation of the AGE-RAGE axis also evokes inflammatory and thrombogenic reactions in ECs by inducing MCP-1, intercellular adhesion molecule-1, and plasminogen activator inhibitor-1 expression via ROS generation [67-73]. Several lines of evidence implicate VEGF as the key factor involved in tumor growth and metastasis [74]. VEGF expression levels are associated with angiogenesis and macrophage infiltration, the extent of which being correlated with various tumor prognoses [75-79]. So, the AGE-RAGE interaction could stimulate tumor-associated angiogenesis and macrophage infiltration by inducing VEGF expression and its related inflammatory reactions, thereby promoting tumor growth and metastasis. In addition, the AGE-RAGE-evoked thrombogenic reactions could cause ischemia and hypoxia within the tumor environments and trigger VEGF expression again, thus further augmenting tumor angiogenesis and inflammation. Further, hypoxia has been known to stimulate brisk generation of AGEs by ECs and subsequently activates the RAGE downstream pathway, which could induce up-regulation of Egr-1, thereby causing inflammatory and thrombotic reactions within the hypoxic areas of tumors [80]. Therefore, these positive feedback loops between AGE-RAGE axis and hypoxia might further potentiate tumor-associated angiogenesis and inflammation, being involved in HCC growth and expansion.

HCC lines resistant to hypoxia were found to have higher levels of RAGE expression, and RAGE overexpression showed significantly prolonged survival under hypoxia [81]. Furthermore, cytoplasmic expression of RAGE was correlated with poorly differentiated HCC, and RAGE was an independent predictor for both overall survival and disease-free survival in patients with HCC after hepatectomy [82].

Knockdown of RAGE by small interfering RNAs inhibited proliferation of HuH7 cells and induced G1 arrest of this cell type, whereas HMGB1, one of the ligands for RAGE, exerted opposite effects on HuH7 cells [83]. HMGB1-RAGE-evoked NF-κB activation has been shown to promote the invasiveness of HCC via activation of heat shock protein 70 [84]. Ethyl pyruvate induced apoptosis and cell cycle arrest in G phase in HCC by suppressing the HMGB1-RAGE-induced Akt activation and matrix metallopeptidase-9 expression [85]. Moreover, in hypoxic HCC cells, HMGB1 activates the RAGE-signaling pathway to induce caspase-1 activity with subsequent production of multiple inflammatory mediators, which, in turn, could enhance the invasion and metastasis of HCC [86].

## Conclusions

**Figure 1:**
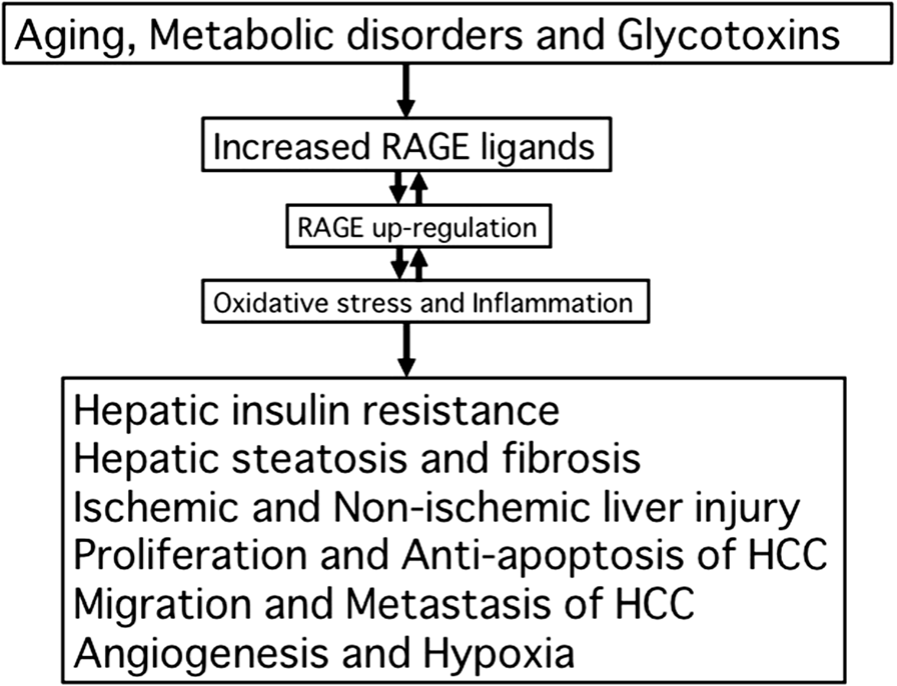
**Role of RAGE in various hepatic disorders.**

As discussed here, there is accumulating evidence that RAGE could play a pathological role in numerous liver diseases via increased oxidative stress generation and inflammatory reactions (Figure 1). RAGE ligands up-regulate RAGE expression itself in the liver, further potentiating the harmful effects of RAGE ligands on hepatic insulin resistance, steatosis, and fibrosis, ischemic and non-ischemic liver disease, and growth and metastasis of HCC, especially in elderly persons or patients with metabolic disorders. Blockade of the RAGE downstream pathway, knockdown of RAGE expression, or restriction of dietary AGEs might be a novel therapeutic target for these devastating hepatic disorders.
